# Design of Ultrasonic Synthetic Aperture Imaging Systems Based on a Non-Grid 2D Sparse Array

**DOI:** 10.3390/s21238001

**Published:** 2021-11-30

**Authors:** Júlio Cesar Eduardo de Souza, Montserrat Parrilla Romero, Ricardo Tokio Higuti, Óscar Martínez-Graullera

**Affiliations:** 1 Faculdade de Engenharia, Campus Ilha Solteira, Universidade Estadual Paulista (UNESP), Avenida Brasil, 56, Ilha Solteira 15385-000, SP, Brazil; julio.c.souza@unesp.br (J.C.E.d.S.); ricardo.t.higuti@unesp.br (R.T.H.); 2 Instituto de Tecnologías Físicas y de la Información (ITEFI-CSIC), C/Serrano 144, 28006 Madrid, Spain; m.parrilla@csic.es

**Keywords:** sparse array, synthetic aperture imaging, ultrasonic imaging

## Abstract

This work provides a guide to design ultrasonic synthetic aperture systems for non-grid two-dimensional sparse arrays such as spirals or annular segmented arrays. It presents an algorithm that identifies which elements have a more significant impact on the beampattern characteristics and uses this information to reduce the number of signals, the number of emitters and the number of parallel receiver channels involved in the beamforming process. Consequently, we can optimise the 3D synthetic aperture ultrasonic imaging system for a specific sparse array, reducing the computational cost, the hardware requirements and the system complexity. Simulations using a Fermat spiral array and experimental data based on an annular segmented array with 64 elements are used to assess this algorithm.

## 1. Introduction

The development of real-time ultrasonic imaging systems based on arrays is a complex issue that encompasses different fields of study, such as material science [1], manufacturing [2], physics [3], and electronic integration [4]. Signal processing also plays a fundamental role in this challenge [5,6], mainly to establish a good trade-off between hardware complexity and image requirements.

Synthetic Aperture Focusing Techniques (SAFT) allow the system designer to reduce hardware requirements at the expense of reduced image frame rate [5,6,7,8,9]. A synthetic aperture imaging system is based on a two-step process: data acquisition and beamforming. The complexity and necessary resources associated with both processes are determined by what we call here the acquisition strategy (ACQ). The data acquisition sub-process follows this strategy, which is based on the independent capture of the signals that correspond with each emission/reception pair (e-r pair) for the selected aperture. The most complete acquisition strategy is the Full Matrix Capture (FMC), which, for an *N*-element aperture, captures all the (N×N) signals (one for each e-r pair). Then, the beamforming sub-process performs the compensation of emission and reception delays at each point of the Region Of Interest (ROI) using the Total Focusing Method (TFM) [9], producing the value of the ultrasound reflectivity at each point $\vec{x}$ as:

(1) $$A\left(\vec{x}\right)=\left|\sum_{i=1}^{N}\sum_{j=1}^{N}s_{ij}\left(t\right)\delta \left(t-(\tau_{i}\left(\vec{x}\right)+\tau_{j}\left(\vec{x}\right))\right)\right|,$$

where $s_{ij}\left(t\right)$ is the signal received by element *j* when *i* is the emitter, $\tau_{i}\left(\vec{x}\right)$ and $\tau_{j}\left(\vec{x}\right)$ are the time-of-flights from elements *i* and *j* to point $\vec{x}$, respectively, and δ(t) is the Dirac delta function.

Although the TFM generates a high-quality image, frequently named as *gold standard*, the FMC offers a poor trade-off between hardware parallelism and frame rate. The ratio between the number of signals (N×N) and the number of parallel electronic reception channels determines the number of acquisition operations. As a result, low parallelism in reception increases the acquisition time. Furthermore, this large number of signals increases considerably the computational cost to generate the TFM image [9,10].

The emission/reception array operation can be modelled as a sampling grid known as coarray [6], and the analysis of its shape is useful to estimate the dynamic range and lateral resolution of the imaging system [6,11]. The emitter/receiver element combination corresponds to a spatial frequency [12], and signals that occupy the same spatial frequency in the coarray are redundant for the beamforming process. The weight of each coarray element is the number of emitter/receiver pairs that are coincident at the same position/spatial frequency. In this sense, in a matrix array, the FMC has a high degree of redundancy and if redundant information is eliminated, in exchange for a reduction in signal to noise ratio, we can reduce the computational cost and simplify the acquisition process [6,12,13].

For matrix apertures, this analysis provides a straightforward way to simplify system acquisition design [5,6,7]. The most efficient coarray is the Minimum Redundancy Coarray (MRC), where just one element occupies each spatial frequency or coarray location [14]. Such a solution, using the minimum number of signals, provides the maximum degrees-of-freedom, better lateral resolution and avoids grating lobes. Furthermore, depending on the available hardware/electronic resources, it can be achieved with different acquisition strategies [12,15].

Non-grid arrays (e.g., spiral or circular distributions), due to their good performance, are becoming very popular [16,17,18,19,20,21,22]. Recently, in [23], synthetic aperture imaging techniques were developed and experimentally tested for a random array. However, the irregular geometry of non-grid arrays makes redundancy identification a complex issue, and there is a lack of adequate tools to study how redundancy is organized on them and how it can be used to improve the imaging system design, and our contribution fills this gap in this important research and practical application area.

Compared to sparse matrix arrays, sparse non-grid arrays allow better optimisation of the number of transducers. Regardless, they also show significant levels of redundancy, but, except for the reciprocity principle ($s_{ij}\left(t\right)\equiv s_{ji}\left(t\right)$) [24], it is not easy to identify other redundancies. Although a reduction in the number of signals has been proposed by random selection of a subset of elements operating as emitters [23], the study of how redundancy is structured and how it can be used to generate a less-redundant acquisition strategy is the key to design more efficient imaging systems with fewer resources.

**Figure 1:**
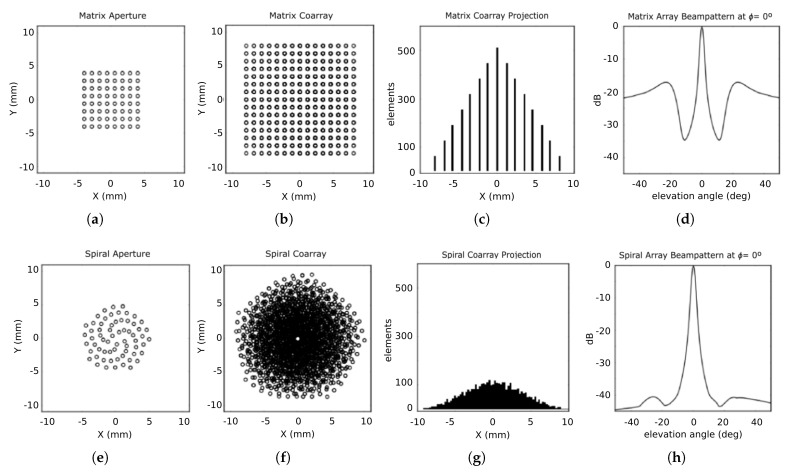
For Matrix arrays: (**a**) array footprint or aperture; (**b**) coarray footprint; (**c**) equivalent linear array projection at $\phi =0^{{}^{\circ}}$, and (**d**) beampattern at $\phi =0^{{}^{\circ}}$ (wideband response, BW = 60%). For spiral array aperture: (**e**) array footprint; (**f**) coarray footprint; (**g**) equivalent linear array projection at $\phi =0^{{}^{\circ}}$, and (**h**) beampattern at $\phi =0^{{}^{\circ}}$ (wideband response, BW=60%).

The differences between matrix and non-grid arrays are presented in  Figure 1a, for a matrix array, and in  Figure 1e for a spiral array. Both arrays have the same number of elements (N=64), and the same dimensions (side or diameter D=16λ, where λ is the wavelength). The matrix array generates a regular coarray of 2λ-spacing grid ( Figure 1b), while the spiral array generates a coarray with an irregular pattern that has smaller and irregular spacing between elements ( Figure 1f). The coarray element distribution has consequences on the beampattern (calculated for wideband response, −3 dB bandwidth BW=60%) as it influences the number of constructive interferences in the sidelobe region. This can be observed in the beampatterns shown in  Figure 1d for the matrix array and  Figure 1h for the spiral aperture, where the different characteristics of mainlobe width and sidelobe levels can be observed.

In this work, we propose the use of the Radon transform and the Projection-Slice theorem to identify the redundancy in the coarray of non-grid apertures. This theorem states that the Fourier transform of the projected coarray in an angle ϕ is identical to the two-dimensional Fourier transform of the coarray evaluated along a slice at the same ϕ [25]. This projected coarray is known as the equivalent linear array at angle ϕ [26].  Figure 1c illustrates an equivalent linear array for the matrix array, and   Figure 1g for the spiral array, both for $\phi =0^{{}^{\circ}}$. Meanwhile the equivalent linear array of the matrix array has a high degree of coincidence in its elements location and concentrates them in very few locations, producing a triangular shape, in the spiral array the coarray elements are less coincident and the equivalent linear array is denser and approximately Gaussian-shaped.

In this work, these concepts are explored to study the coarray spatial redundancy of non-grid 2D sparse arrays like spirals or annular rings, so that these arrays can be designed by using objective parameters. Additionally, the detailed analysis of spatial redundancy information yields a reduction in the electronic resources and the computational cost of the beamforming operations of the synthetic aperture system.

Section 2 analyses basic characteristics of arrays and the involved redundancies. Section 3 describes how to assess redundancy information to design acquisition strategies, and Section 4 illustrates two cases involving non-grid arrays: Fermat spiral and segmented annular arrays. Section 5 presents some discussions and Section 6 the conclusions.

## 2. The Coarray Analysis

### 2.1. Acquisition Strategies

Consider an array where the transmitting and the receiving apertures are represented by the sets $X_{E}$ and $X_{R}$. Assuming that the position of each element on the XY plane is given by a vector ${\vec{e}}_{i}=\left(x_{i},y_{i},0\right)$, the set of coarray elements, *C*, is obtained from:

(2) $$C=\left\{{\vec{c}}_{ij}={\vec{e}}_{i}+{\vec{e}}_{j}\right\}\;\forall \;{\vec{e}}_{i}\in X_{E},\;{\vec{e}}_{j}\in X_{R},$$

where ${\vec{c}}_{ij}$ is a coarray element generated by the emitter *i* and the receiver *j*.

**Figure 2:**
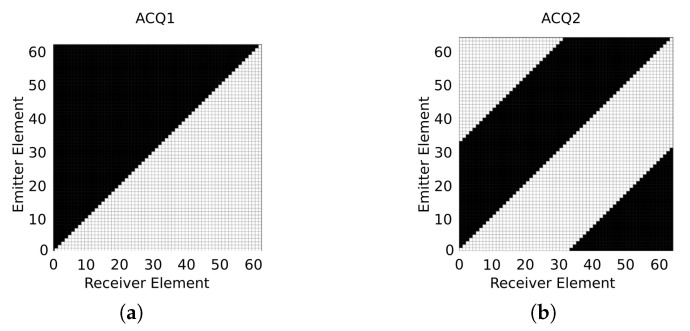
Matrix representation of acquisition strategy (ACQ) for a generic array composed by 64 elements. White cells are the active channels. (**a**) ACQ1—the reciprocity principle has been applied to reduce the number of signals. (**b**) ACQ2—further optimisation of the electronic resources.

Each emitter/receiver pair and its associated signal $s_{ij}\left(t\right)$ correspond to a unique coarray element. By the reciprocity principle, it is easy to identify that ${\vec{c}}_{ij}$ and ${\vec{c}}_{ji}$ introduce equivalent information in the beamformer. By extending this principle, an acquisition strategy that reduces the number of signals by almost half can be used [24,27].  Figure 2a illustrates this strategy (ACQ1), where each white square corresponds to one acquired signal. Moreover, the acquisition strategy ACQ1 can be rearranged to create the ACQ2, shown in  Figure 2b, which allows further reduction in the number of parallel channels by almost twice.

**Figure 3:**
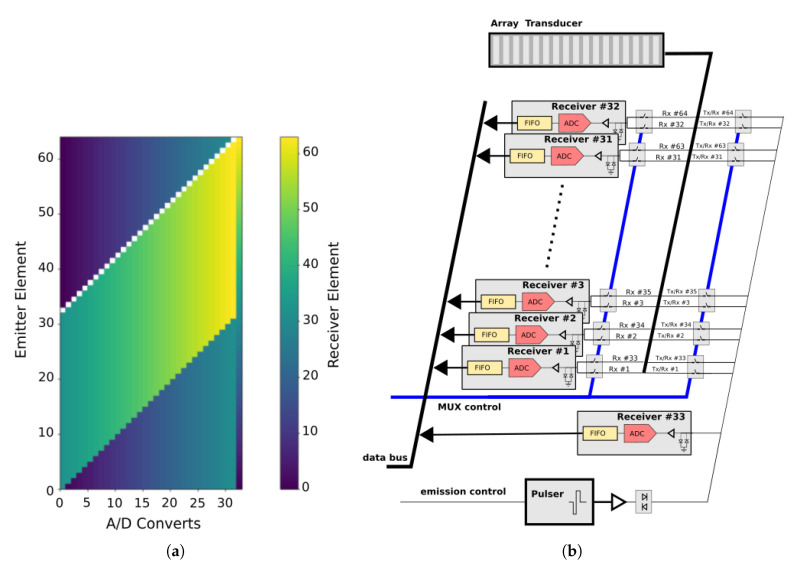
(**a**) Compact representation of ACQ2 and (**b**) a corresponding ultrasonic acquisition system.

Using an array with 64 elements involves 64×64=4096 signals in the FMC. By excluding the pulse-echo signals, there are 4096−64=4032 signals, and using the reciprocity principle, $s_{ij}=s_{ji}$, and only 4032/2=2016 signals are needed for the different emitter/receiver pairs. Summing the 64 pulse-echo signals, results in 2080 signals. In  Figure 2b, ACQ2 strategy reduces the number of signals involved from 4096 to 2080, and the number of receiver parallel channels from 64 to 33. This structure can be reorganised to obtain a simpler implementation, and   Figure 3a illustrates a more compact representation of ACQ2. Reciprocity allows to empty less than a half of the matrix, and we can make a rearrangement of the columns to reduce the number of converters. The acquisition strategy is organized in a matrix representation, where each shot (associated with a transmitter element) is represented in a row, and each column is tied to a specific converter in reception. Each colour of the cells indicates the number of the receiver transducer. As it can be seen, the last converter channel (#33) handles the pulse-echo signals ($s_{ii}\left(t\right)$), which means that, for this converter, 63 receiver changes will take place. For the other converters, we fill each column with the respective receiver to implement the acquisition strategy ACQ2. For example, column 1 (converter 1) is filled at the bottom with the receiver 33 and changes to receiver 1 after the emitter 33. Using this strategy, the 32 first receivers share each one only two different elements, and the multiplexer is simplified. As converter #33 needs to access all transducers, in order to avoid an extra multiplexing net, this channel can share the multiplexing structure with emission. In this implementation, there are switched-off receivers (white cells in  Figure 3a) that occur because converter #33 is in charge of these acquisitions.

In  Figure 3b, there is a model of the ultrasonic system based on this acquisition strategy. In this case, the multiplexer is implemented as two independent nets controlled by 64 switches each. In one net, each receiver channel, from 1 to 32, is associated with two transducers (the i−th and (32+i)−th). In the other net, the 64 switches share the connection between the pulser (emitter) and the #33 receiver circuit.

It is important to highlight that reciprocity does not depend on how the elements are distributed, and it can be applied to linear and bidimensional configurations.

### 2.2. Identification of Redundancy in the Coarray

Redundancy is produced when a coarray position is filled by more than one e/r element. In matrix arrays, redundancy is easily identified because the coarray elements are organised in a regular grid. However, in non-grid distributions, once the reciprocity principle is used, the positions of the coarray elements are rarely coincident (see  Figure 1b), so most of them should be considered as non-equivalent or non-redundant. Nevertheless, some coarray elements could be considered equivalent for the beamforming process if there is a minimum distance between them. This minimum distance can be evaluated by using the focusing delay quantification theory [28,29,30], which states that errors introduced by the quantification of focusing delays can be rejected as long as they remain below $(\frac{\lambda }{32})/c$, where *c* is acoustic propagation velocity. Then, if the distance between two coarray elements is less than $\frac{\lambda }{32}$, we can consider that they introduce fundamentally the same information in the beamforming process. However, if sparsity is high, the redundancy revealed by this clustering operation is very low, and a useful alternative, proposed here, is to study how redundancy is organised in the equivalent linear array.

### 2.3. Identification of Redundancy in the Equivalent Linear Array

From the Projection-Slice theorem [31], the narrowband beampattern of a 2D array in a particular azimuth direction is determined by the projection of all the coarray elements on an axis along the same direction. Consequently, two elements ${\vec{c}}_{i_{1}j_{1}}$ and ${\vec{c}}_{i_{2}j_{2}}$ are considered redundant in a given angle ϕ if:

(3) $$|\langle {\vec{c}}_{i_{1}j_{1}},\left(\sin\phi ,\cos\phi \right)\rangle -\langle {\vec{c}}_{i_{2}j_{2}},\left(\sin\phi ,\cos\phi \right)\rangle |\leq \frac{\lambda }{32},$$

where <·> is scalar product.

**Figure 4:**
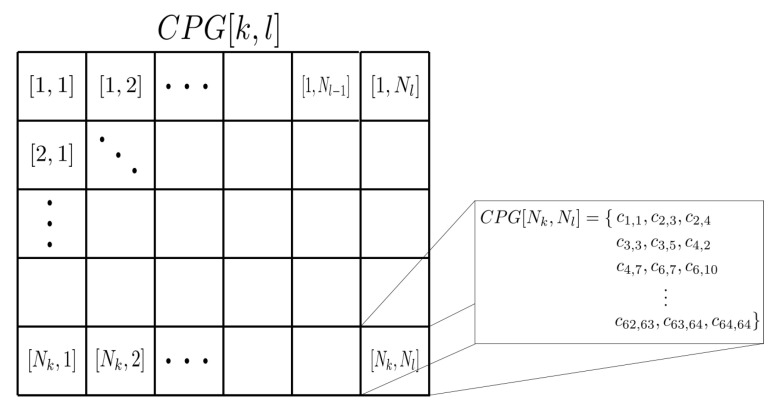
Example of Coarray Projection Grid (CPG) where each [k,l] position contains a set of coarray elements $c_{ij}$ that meet the condition (4).

Although this is a specific solution for each azimuth angle, if the whole azimuth domain is considered, we can generate a map showing the redundancy relationship between the different coarray elements along the azimuth angle. Essentially, this map is an unstructured Radon transform where the projected elements can be clustered along the azimuth and radial axis. This information can be sampled in a grid structure that we have named Coarray Projection Grid (CPG). The centre of the CPG elements is located using the Nyquist criterion, which establishes that the resolution in the radial dimension is given by (λ/2) and in the angular axis it is defined by the mainlobe width of the coarray. Then,

(4) $$CPG\left[k,l\right]=\left\{c_{ij}\right\}\;\forall \;\left|\langle {\vec{c}}_{ij},\left(\sin\left(k\phi_{K}\right),\cos\left(k\phi_{K}\right)\right)\rangle -l\frac{\lambda }{2}-D\right|<\frac{\lambda }{32},$$

where the angular discretization is given by $\phi_{K}$:

(5) $$\phi_{K}=\frac{1}{2}\arcsin\frac{\lambda }{2D},$$

and CPG is an $\left(N_{k}\times N_{l}\right)$ matrix of sets ( Figure 4), where:

(6) $$N_{k}=\left\lceil \frac{2\pi }{\phi_{k}}\right\rceil \;and$$

(7) $$N_{l}=\left\lceil \frac{2D}{\lambda /2}\right\rceil +1.$$

Elements out of the grid are considered oversampling elements. The CPG can be used to study the redundancy of any acquisition strategy and describes the potential of an aperture to be optimised. Two parameters can be obtained from it: the occupancy rate of the CPG ($M_{o}$), that is the percentage ratio between occupied locations and the total number of possible locations, and the redundancy level ($M_{r}$), which is the mean number of signals per occupied CPG location, and it indicates the level that an optimisation process can be performed.

## 3. Synthetic Imaging System Design

We define a particular acquisition strategy ACQ as a list of e-r pairs used to obtain the data set. To evaluate its performance, we can use $M_{o}$, $M_{r}$ and the number of signals involved ($N_{s}$). For the ideal ACQ, all CPG positions should be filled with only one coarray element ($M_{o}=100\%$ and $M_{r}=1$), meaning that the beampattern generated by this ACQ has the maximum lateral resolution, as well as equivalent distribution of side lobes at each azimuth angle. In the case of a sparse array, the CPG is sparsely and irregularly filled and it is difficult to achieve the ideal ACQ. However, we can use it to reduce the number of signals involved by eliminating overlapping coarray elements at each CPG[k,l], while the occupancy $M_{o}$ is maintained.

To make programming tasks easier, we propose to reorganise the CPG structure in another (N×N) matrix. Each [i,j] position of this new matrix, named Inverse Coarray Projection Grid (ICPG), corresponds to the coarray element $c_{ij}$ and, inside each position of this matrix, the different [k,l] values that $c_{ij}$ satisfy using the condition (4) will be stored.

The process to create the ICPG database is defined in Algorithm 1.
**Algorithm 1:** Generation of the ICPG database1:**for***i***in range(***N***)**:2:     **for** *j* **in range(***N***):**3:        ICPG[i,j]=[]4:        **for** *k* **in range(**$N_{k}$**):**5:            $C_{ij}\left(k\right)=\langle {\vec{c}}_{ij},\left(\sin\left(k\phi_{k}\right),\cos\left(k\phi_{k}\right)\right)\rangle$     projected element6:            **for *l* in range($N_{l}$):**7:               **if $\left(\left|C_{ij}\left(k\right)-l\frac{\lambda }{2}-D\right|<\frac{\lambda }{32}\right)$**8:                   ICPG[i,j]**.append(**[k,l]**)**9:**return** CPG                          database

The ICPG facilitates the evaluation of the relevance of a specific element on the acquisition strategy and the introduction of operation restrictions in the system design (like avoiding particular broken elements).

### Acquisition Signals Selection

The acquisition strategy optimisation is described in the Algorithm 2, where the ACQ (acquisition strategy) is a list of $G_{c}=\left[i,j\right]$ values that defines the pair elements used in acquisition process(emitter *i* and receiver *j*). This algorithm depicts a particular case, where we limit the number of emitters and parallel channels.
**Algorithm 2:** Algorithm for selecting the acquisition strategy(ACQ)1:MAP**= zeros**$\left(N_{k},N_{l}\right)$                          Filling matrix2:ACQ=[]                       initialisation acquisition strategy3:GFP=1, GNFP=1                    Global position counters4:CAN=[]                                 signal sets5:**for***i***in range(***N***):**6:     **for** *j* **in range(***N***):**7:        **CAN**.append**([i,j])**8:EP**= zeros(**2,N**)**                     Element Participation counter9:**while (**GFP>0**):**10:     GFP=0, GNFP=011:     **for** i0 **in** CAN**:**12:        **if** (EP[[0,1],i0]<EPR[0,1])**.all:**            Tx:Rs parallelism check13:            NFP,FP **= compute_FP(**MAP,ICPG[i0]**)**14:            **if** (GFP≤FP)or((GFP==FP)and(NFP<GNFP))**:**15:               GFP=FP; GNFP=NFP;16:               $G_{c}=i0$17:     **if** (GFP>0)**:**18:        ACQ**.append**$\left(G_{c}\right)$19:        CAN**.remove**$\left(G_{c}\right)$20:        $EP[G_{c}\left[0,:\right]]+=1$21:        $EP[G_{c}\left[1,i0\left[1\right]\right]]+=1$22:        **for** i0 **in** $ICPG[G_{c}]$**:**23:            MAP[i0]=MAP[i0]+124:**return** ACQ                           Acquisition Strategy

The contribution of the ACQ to the projected coarrays is analysed in the MAP matrix, which is a counter related to CPG. In this representation of the CPG, it is easier to compute $M_{o}$ and $M_{r}$. The proposed heuristic selects the emission/reception configuration (ACQ) that best fills the MAP matrix with the minimum number of signals. Specific conditions, such as eliminating specific elements, can be introduced when the content of ICPG is changed. Furthermore, by introducing specific counters the number of receivers per emission can be limited (counters of Element Participation, EP, and Element Participation Restriction, EPR, defined as EPR=(emission:reception)), and other conditions can be introduced, like using different elements for emission and reception.

The algorithm initialises the MAP matrix, the EP, and the sequence CAN of emission/reception pairs. In the case where there are no restrictions, CAN is initialised with all combinations of emitters and receivers (FMC). After that, there is a loop, where the contribution MAP of each coarray element (CAN[i0]=[i,j]) is evaluated by how much information is introduced (the number of positions that are filled where MAP[k,l]=[], FP counter) and by how much redundancy is introduced (positions where MAP[k,l]≠[], NFP counter). The process is controlled by Global Free Positions (GFP) and Global Non-Free Positions (GNFP), that are the counters of the candidate ($G_{c}$) to be incorporated to ACQ. If the emitter *i* and receiver *j* have free parallel channels ((EP[[0,1],i0]<EPR[0,1])**.all**:), and the pair [i,j] contributes with more information (FP>GFP) and less redundancy (NFP<GNFP) to the MAP, it becomes a candidate ($G_{c}=i0$) and GFP and GNFP are updated. At the end of the loop, the candidate is appended to ACQ, the MAP and CAN are updated and a new search begins with the remaining elements, until GFP=0, which means that the sequence cannot fill more new MAP positions and adding more elements would increase redundancy.

## 4. Design of Acquisition Strategies: Simulation and Experimental Results

Two different arrays were chosen to evaluate the performance of the proposed technique. The first is a 64-elements Fermat spiral array [16], where simulation was used to analyse it. The second is a 64-elements segmented annular array [32], where simulation and several experimental ultrasonic images were generated to verify the results. The proposed algorithm was evaluated by considering the beampattern, the MAP occupancy level ($M_{o}$), the redundancy level ($M_{r}$) and the number of signals involved ($N_{s}$).

Our simulation model is based on the implementation of the spatial impulse response, following the method proposed in [33]. This model allows a wideband analysis of the beampattern and, for simplicity, the elements were considered as point sources. The beampattern is simulated in a semi-sphere ($\theta \in [-90^{{}^{\circ}}$:Δα:$+90^{{}^{\circ}}]$, $\phi \in [0^{{}^{\circ}}$:Δα:$180^{{}^{\circ}}]$, $\Delta \alpha =\frac{1}{2}\Delta \theta_{-6\mathrm{dB}}$), where $\Delta \theta_{-6\mathrm{dB}}$ is the −6 dB mainlobe width, in degrees. From the semi-sphere, three lateral profiles are composed by obtaining, at each elevation angle, the maximum, the mean and the minimum values. In the following figures, for comparison between the FMC and the examined designed strategy (ACQ), their acoustic field lateral profiles are presented. The light grey areas (contoured by dashed lines) show the result of the ACQ, whereas dark grey areas (contoured by solid lines) are related to the FMC. Furthermore, an inset shows a detail of lateral resolution for small elevation angles.

### 4.1. Fermat Spiral Array—Simulation Results

**Figure 5:**
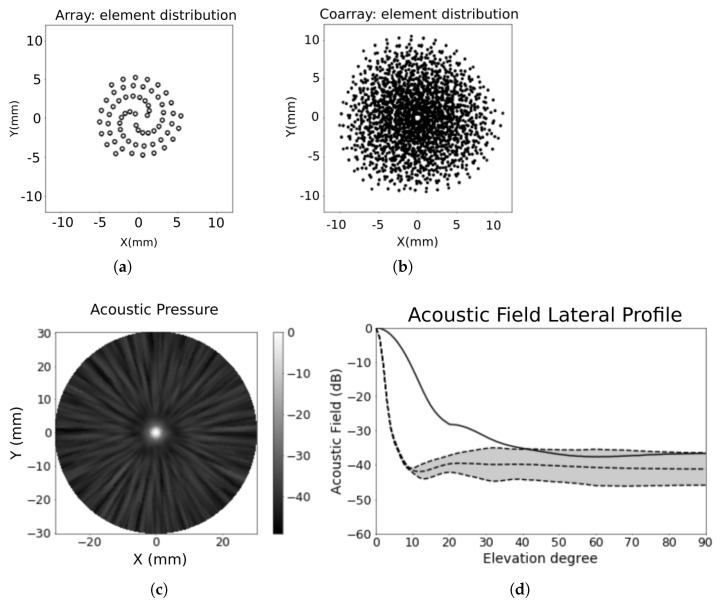
The 64-elements Fermat spiral array. (**a**) Array footprint; (**b**) coarray footprint; (**c**) acoustic pressure in the semi-sphere [$\theta =0^{{}^{\circ}}$:$90^{{}^{\circ}},\phi =0^{{}^{\circ}}$:$360^{{}^{\circ}}$] and (**d**) lateral profile showing the distribution of the sidelobes in elevation. The dashed lines represent the spiral beampattern, and the light grey area within that lines shows sidelobe distribution at each elevation angle. The solid line is the corresponding beampattern of a 64-elements matrix array (8×8 elements).

The Fermat spiral array was designed with a diameter of 22λ, 64–elements and a divergence angle $\alpha =125.764^{{}^{\circ}}$, central frequency $f_{c}=3.0$ MHz, BW=60%, operating in water (c=1500 m/s) and focused at 60 mm. The aperture and its beampattern are shown in Figure 5: (a) array footprint, (b) coarray structure, (c) acoustic pressure, and (d) lateral profile of the acoustic pressure at each elevation angle (light grey area contoured by the dashed line).

The beampattern response of a 64-element matrix array (8×8), with its elements spaced by (λ/2), is also illustrated in Figure 5d with a solid line. The matrix array has a similar dynamic range (−30 dB) and worse lateral resolution than the spiral array. The spiral distributions aim to spread the energy in large sidelobes regions, avoiding a high concentration of energy [16,17]. In this case, the mean sidelobe level for the spiral array is around −40 dB with ±5 dB deviation.

By clustering the coarray elements that are less than (λ/32) apart, only six signals $s_{ij}$ are added to the set of redundant signals (initially made up of reciprocal elements). Using the projections, the FMC occupies 40,422 positions of the possible 49,770 in the CPG structure; that results in $M_{o}=81\%$ and $M_{r}=7.23$. If the reciprocity principle is used to create an ACQ, which is named RCP (see Table 1), $M_{r}$ is reduced to 3.68, which still indicates a very redundant distribution. Additional results are summarised in Table 1, where $N_{s}$ is the number of signals selected and $N_{c}$ is the maximum number of parallel channels for each strategy in reception. Besides FMC, reciprocity reduction (RCP) parameters have also been included. The other parameters will be introduced in the following paragraphs.

**Figure 6:**
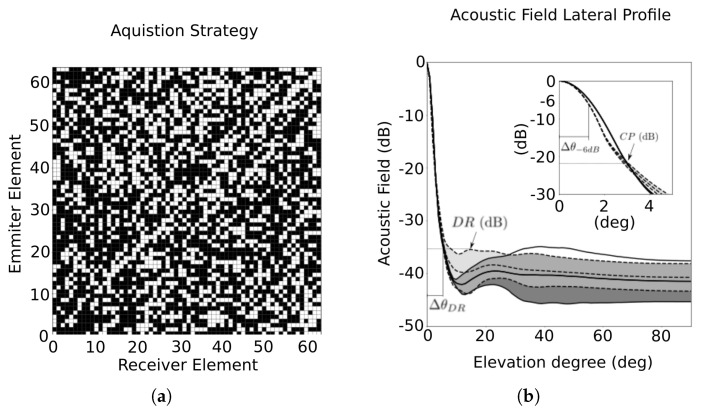
(**a**) Strategy ACQ(64:64) for redundancy reduction. (**b**) Sidelobe distribution at each elevation angle for strategy ACQ(64:64) (light grey area within the dashed lines) and the FMC (dark grey area within the solid lines).

Then, using the proposed heuristic, the first acquisition strategy, ACQ(64:64) is illustrated in Figure 6. In this example, the Element Participation Restriction (EPR) is defined by using 64 emitters and 64 parallel channels ($T_{x}$:$R_{x}=64$:64) and the signals that are considered redundant are eliminated. Comparing to the FMC, we achieved the same $M_{o}$ with only 39% of the signals and a 59% reduction in $M_{r}$. Also, it has reduced the number of reception channels. The mean number of reception channels per emission is 25, with a maximum of 32 and a minimum of 14. Furthermore, it employs 450 fewer signals than the RCP. Comparing the acoustic field of (64:64) with the original aperture, we see that the sidelobe distribution has a smoother distribution with an overall increase of only 2 dB in comparison to the FMC.

Also in Figure 6b, four additional parameters are shown: DR is the Dynamic Range (dB), defined as the maximum sidelobe level relative to the mainlobe level; $\Delta \theta_{DR}$ (degrees) is the mainlobe width, defined at the level of DR; Cross-Point, CP (dB), is defined by the level that the ACQ lateral profile crosses the FMC lateral profile; and the aforementioned $\Delta \theta_{-6\mathrm{dB}}$ is the −6 dB mainlobe width. It can be seen that the ACQ(64:64) lateral resolution is higher than FMC’s until CP=−21 dB. The dynamic ranges are similar, and $\Delta \theta_{DR}$ increases a bit, although the value of $\Delta \theta_{-6\mathrm{dB}}$ is smaller for the ACQ(64:64), as can be observed in Table 1.

**Figure 7:**
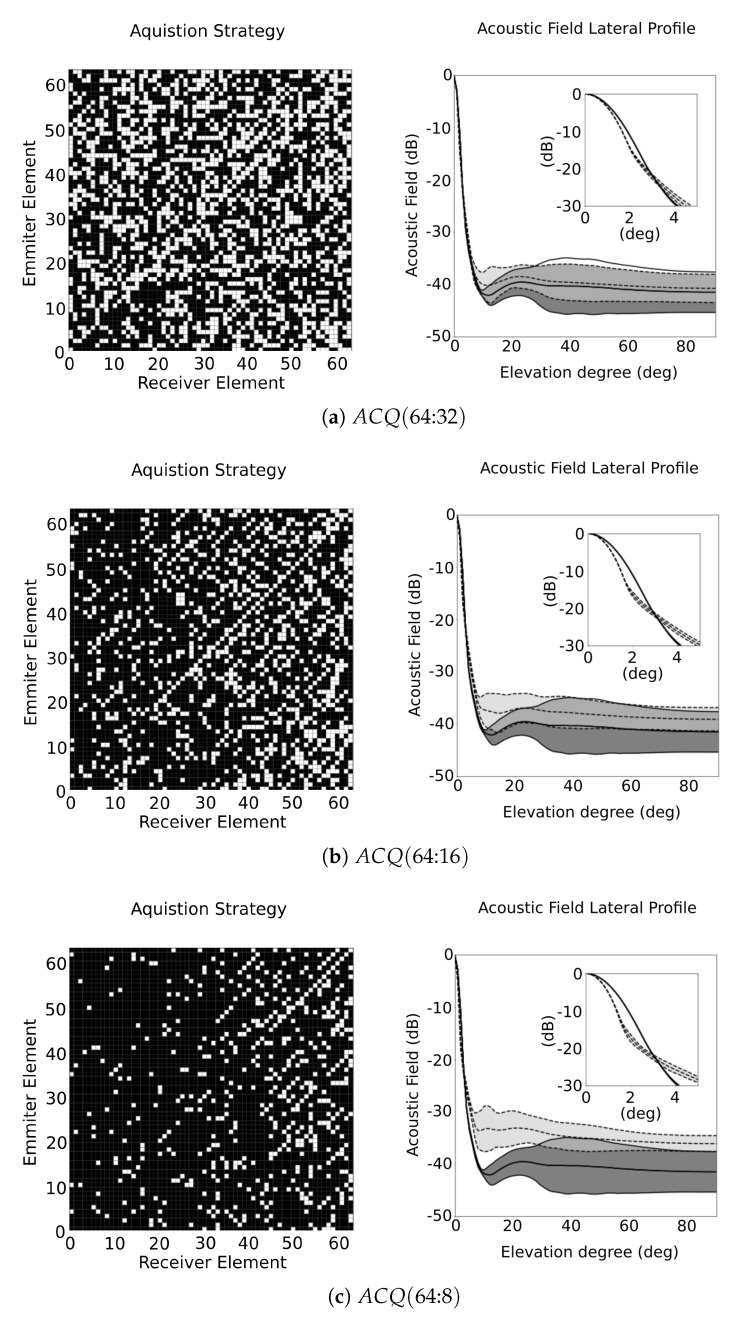
ACQ obtained from the strategy and sidelobes distribution at each elevation angle for strategies (**a**) ACQ(64:32), (**b**) ACQ(64:16) and (**c**) ACQ(64:8), where the distribution of the respective ACQ is represented in light grey area within the dashed lines, and the FMC is represented as dark grey area within the solid-lines.

The algorithm can be adapted to limit the maximum number of emissions and receptions. To illustrate this, the following EPR values are defined: ACQ(64:32), ACQ(64:16), ACQ(64:8), ACQ(32:64) and ACQ(16:64). The first three strategies defines that all 64 emitters can be used and the restriction of the algorithm resides in the number of parallel receivers, with a significant restriction in the ACQ(64:8) case. The results obtained from the first three settings are illustrated in Figure 7a–c, where it is possible to see the matrix representations of acquisition strategies and the distribution of the lobes in the beampattern at each elevation angle.

In ACQ(64:32) ( Figure 7a), the results are similar to ACQ(64:64) (See also Table 1). The number of parallel receivers is the same and, comparing the lateral profiles of ACQ(64:32) and ACQ(64:64) ( Figure 6b), both strategies have almost the same response. One remarkable fact, showing the benefits of the redundancy reduction, is that the response of the FMC has worse lateral resolution in the range of −6 dB and slightly more sidelobes than ACQ(64:64) ( Figure 6b) and ACQ(64:32) ( Figure 7a).

In ACQ(64:16) ( Figure 7b), this configuration has vacant positions in the CPG ($M_{o}$ decreases to 77%). Although there is a small increase in all lateral profiles, and comparing to the FMC response, the sidelobes remain in a similar range. In ACQ(64:8) ( Figure 7c), there is a significant resource reduction by using only 12% of the available signals. In consequence, the sidelobes are 5 dB higher than the FMC response. However, it is remarkable that $\Delta \theta_{-6\mathrm{dB}}$ becomes smaller for ACQ(64:16), even using less resources.

**Figure 8:**
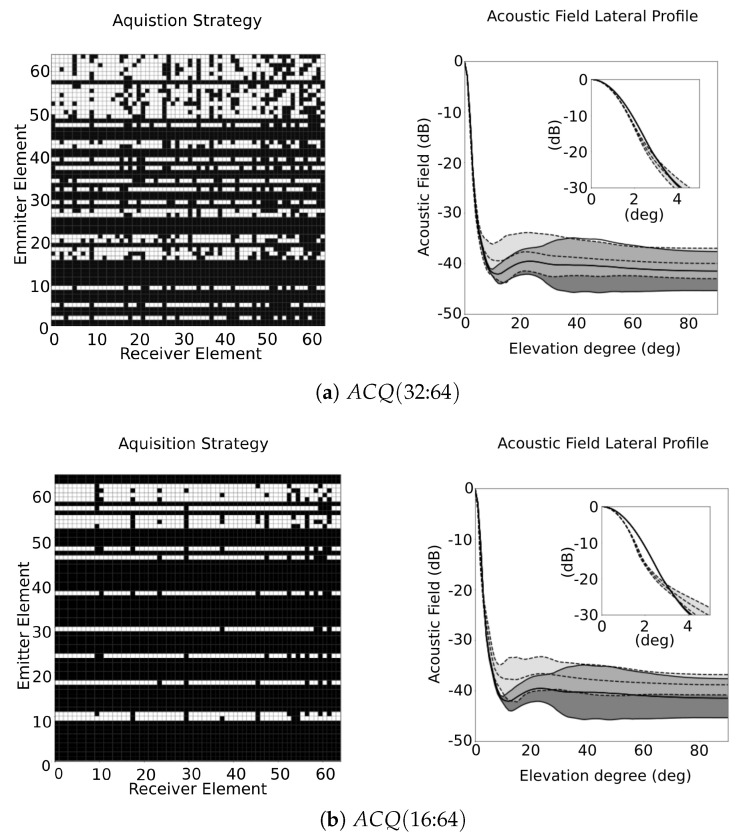
Acquisition strategy and sidelobes distribution at each elevation angle for (**a**) ACQ(32:64) and (**b**) ACQ(16:64), where the distribution of the respective ACQ is represented in light grey area within the dashed lines, and the FMC is represented as dark grey area within the solid lines.

Figure 8 illustrates the results obtained for ACQ(32:64) and ACQ(16:64), where in this case, the number of emitters was limited. The $M_{o}$ values for both cases decrease from 81% to 77% and 65%, respectively. The lateral profile for ACQ(32:64) ( Figure 8a) remains in a similar range comparing to FMC, with a small increase in the mean value, but with smaller variance. Analysing the acoustic response for ACQ(16:64) strategy ( Figure 8b), sidelobes rise above the FMC response and, comparing with ACQ(64:16), which has similar number of signals, it has worse response ( Figure 7b).

Consequently, one important conclusion is that limiting the number of emitters has more consequences in the acquisition strategy than limiting the number of receiving parallel channels. When one emission element is removed from the acquisition, instead of turning off only one position of the ACQ matrix, it will turn off a full line in it, leaving less space for optimisation. On the other side, when the number of emissions decreases, the imaging frame rate increases.

In our examples, the original dynamic range is maintained when the number of resultant signals is higher than 25%. But, when the reduction is more intense, the dynamic range decreases. In the case of the lateral resolution ($\Delta \theta_{-6\mathrm{dB}}$), all of the strategies offer higher values than the FMC and up to −20 dB. From this value, the lateral resolution at the dynamic range is lower than the value of the FMC. This behaviour is a consequence of the smoother shape of the coarray when the reduction is applied.

### 4.2. Segmented Annular Array—Simulated and Experimental Results

#### 4.2.1. Experimental Setup

**Figure 9:**
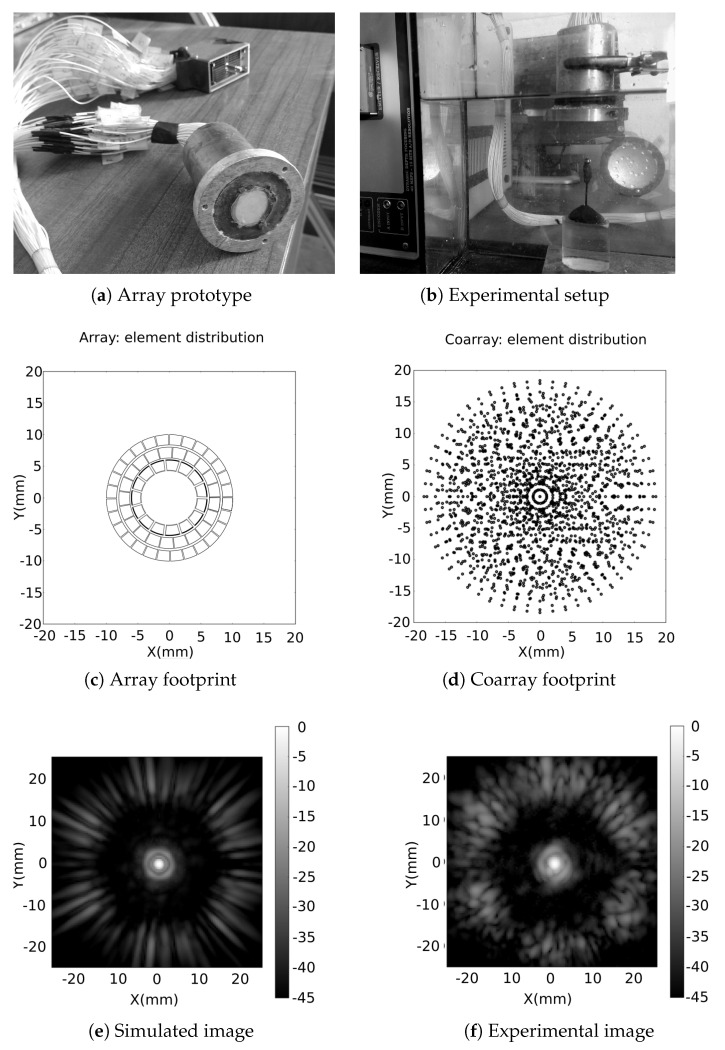
Segmented annular array. (**a**) Array prototype; (**b**) experimental setup in water, array faced downwards and a 3 mm-diameter spherical reflector placed 40 mm from the array; (**c**) element distribution, (**d**) coarray footprint. Image of a (**e**) point reflector (simulated) and (**f**) 3 mm-diameter metallic sphere (experimental) both placed at [X = −25:25 mm, Y = −25:25 mm, Z = 40 mm].

The experimental example is based on the segmented annular array described in [34]. The array prototype is illustrated in Figure 9a, and was manufactured at CSIC laboratory. It has been designed for non-destructive testing (NDT) of metallic parts, but in this paper it is operated in water. The array ($f_{c}=1.5$ MHz, BW=20%) is composed of 64 elements that are organised in three rings, has a 20 mm diameter (in water 20λ), the center of the elements are spaced by 2 mm (in water 2λ) and element size is 1.5×1.5 mm (in water 1.5λ).

Figure 9c illustrates the segmented annular array element distribution and Figure 9d is the corresponding coarray. In order to obtain a more accurate response in our simulation model, the differences in the energy radiated per element and the element radiation pattern were taken into account. The comparison between experimental data and simulations are based on images from a spherical reflector. Figure 9e shows a simulated image of a point reflector and Figure 9f illustrates the experimental image, both set at Z=40 mm, X=[−25:25] mm and X=[−25:25] mm and using the FMC and TFM. With this configuration, in water, experimental results shows a lateral resolution of $5^{{}^{\circ}}$ and a dynamic range, limited by grating lobes at $30^{{}^{\circ}}$, of 20 dB. The differences between simulated and experimental images are due to anomalies in the radiation pattern of the real aperture and the diffraction response of the reflecting sphere. Furthermore, this probe shows significant variations in element-to-element sensitivities, reaching up to 4 dB, which increases the sidelobes level intensities.

#### 4.2.2. Synthetic Aperture Strategies

**Figure 10:**
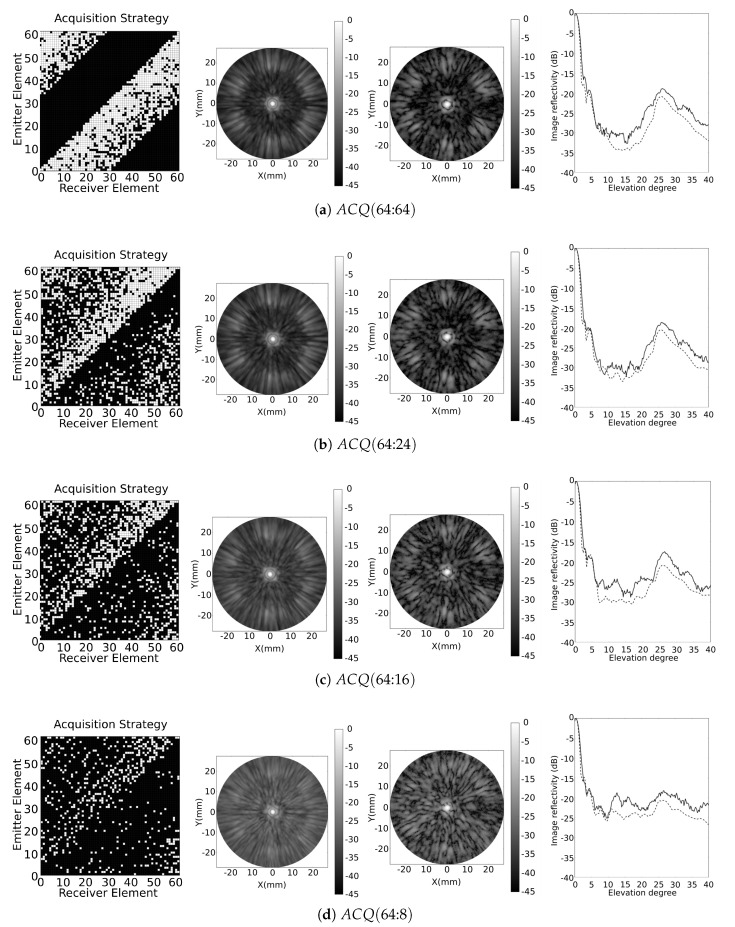
Results for the segmented annular array obtained from ACQ (**a**) (64:64), (**b**) (64:24), (**c**) (64:16) and (**d**) (64:8), where the acquisition strategy, the simulated point reflector, the experimental metallic sphere image (cylindrical coordinates: Z=40 mm R=[0:25] mm, $\theta =[0^{{}^{\circ}}:360^{{}^{\circ}}]$), and the image reflectivity (maximum at each elevation angle) are illustrated, respectively. For the image reflectivity, the simulated response is illustrated by a dashed line and the experimental result by a solid one.

After clustering the coarray elements, 109 signals meet the (λ/32) condition. The CPG matrix has 10,542 positions ($N_{k}=251$ and $N_{l}=42$), and fills 66% ($M_{o}$) of the coarray with $M_{r}=5.45$ elements per coarray position. The RCP configuration works similarly as illustrated in Figure 2. The configurations designed for this example are: ACQ(64:64), ACQ(64:24), ACQ(64:16) and ACQ(64:8). Figure 10 illustrates, for the acquisition strategies: the acquisition matrix, simulated and experimental images of a point/spherical reflector, and a detail of the lateral profile of the maximum beampattern (simulated and experimental). Table 2 summarises several parameters used to evaluate the performance of each strategy.

The ACQ(64:64) and ACQ(64:24) strategies solutions show similar beampatterns, where the sidelobes remained in −30 dB level. For the ACQ(64:16) and ACQ(64:8) strategies, there was an increase of the sidelobes of 5 dB and 10 dB, respectively. We notice that the model predicts the impact of $M_{o}$ reduction, which increases the sidelobes level [35]. Meanwhile, grating lobes, around $28^{{}^{\circ}}$, remained at approximately the same level for all strategies, with a slight increase when Mo is reduced. Mainlobe width was also maintained for all strategies, as observed from $\Delta \theta_{-6\mathrm{dB}}$ and DR (dB) from Table 2.

With respect to the resource optimisation, when compared to the ACQ(64:64), the ACQ(64:24) has a 6% reduction in $M_{o}$, reducing the parallel channels ($N_{c}$) in five, and the number of selected signals ($N_{s}$) by 250. A significant signal reduction (512) is achieved while $M_{o}$ is maintained above 50%, from ACQ(64:64) to ACQ(64:16). The ACQ(64:8) produces a significant reduction in the value of $M_{o}$ (35%), but the secondary lobes increase to the level of the grating lobes, as can be noticed in Figure 10d. There are no dramatic changes in dynamic range (DR) because the array structure is maintained, and is limited by the grating lobes.

## 5. Discussion

The design of a synthetic aperture imaging system should consider a balance between the number of parallel channels, the number of signals in the beamforming, the number of shots to capture all data needed per image, and the multiplexer complexity. In this sense, synthetic imaging beamforming can exploit popular parallel computation resources to obtain 3D imaging at a relatively low cost, using multicore or GPGPU (General-Purpose Graphics Processing Units). In our experience, parallel GPGPU beamforming for 4096 signals can generate a frame rate of 66 images/s (256×256 pixels) [10], being able to generate two volumes per second (128×128×128 voxels). Considering a maximum number of signals ($N_{s}$) of 1024, the GPGPU beamforming can generate up to ten volumes per second. However, to support this volume rate, we need to limit the acquisition operations by reducing the number of emitters and the volume of data transferred per shot to the processing system (parallel receiver channels). Using this information, we analyse, by simulations, two strategies for the Fermat spiral array: ACQ(42:24) and ACQ(32:32).

**Figure 11:**
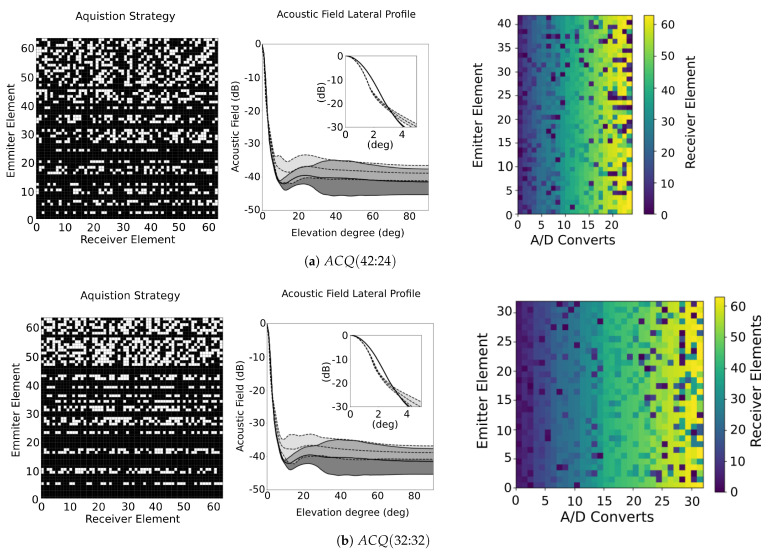
Results obtained from ACQ (**a**) (42:42), and (**b**) (32:32), where the acquisition strategy is presented in the left, the sidelobe distribution at each elevation angle for strategies is illustrated at the centre, and the configuration of the multiplexer net showing, at each shot, the distribution of reception transducer in the reception channels is illustrated in the right. For the sidelobe distribution, the current ACQ is represented in light grey area within the dashed line, and the FMC is represented as the dark grey area within the solid-line.

Figure 11 illustrates the two proposed acquisition strategies and shows the lateral profile of the acoustic field, where dark grey corresponds to the acoustic field of the FMC. Although the acoustic pressure is similar for the two configurations, it is possible to see that the reduction in the number of shots has a worse impact than the reduction of parallel channels in reception.

The results are summarised in Table 3, where FMC and RCP are also listed. In both cases, there was a reduction in $M_{o}$ from 81% to 75% and 74%, respectively. The sidelobe distribution is comparable to the FMC, so, in practice, the image quality is maintained in a similar range. However, the number of signals $N_{s}$ is reduced to less than a half when compared to RCP (2080 to 1008), which means a significant cost reduction in processing. Lateral resolution is also better for the proposed strategies, as can be observed from $\Delta \theta_{-6\mathrm{dB}}$.

The last challenge is the design of the multiplexer net. In our example, the efficient design of the RCP needs up to 128 switches. However, a reduction in the number of receiver channels or/and in the number of emissions has an increment in the number of transducers that has to be attended by each receiver channel (in consecutive shots). As a consequence, the multiplexer net becomes more complex. In Figure 11, the multiplexer matrix is presented for both solutions. The configuration ACQ(42:24) needs 201 switches in reception and 42 in emission. In the case of the configuration ACQ(32:32), it needs 246 in reception and 32 in emission (see $N_{c}$ in Table 3). These nets have been optimized to reduce the number of switches using an *ad-hoc* algorithm, linking transducers to specifics A/D converters. In general we can say that the reduction of parallel resources has a cost in complexity in the multiplexer.

Although the ACQ(42:24) has a significant reduction in the hardware resources (around 25%), the ACQ(32:32) is about 30% faster than ACQ(42:24). Additionally, the analysis of the beampattern, based on the lateral resolution and dynamic range of both ACQ (see Table 3), shows that they have a similar behaviour.

**Figure 12:**
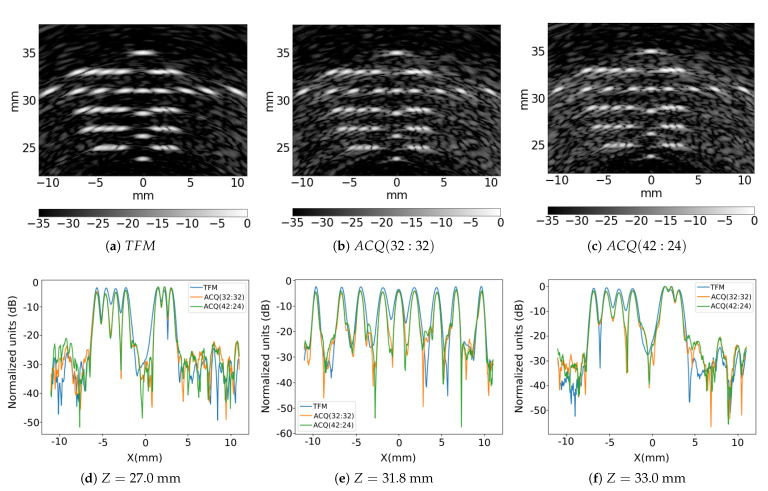
Images of simulated phantom to evaluate ultrasound medical imaging capabilities of the proposed acquisition strategies: (**a**) TFM; (**b**) ACQ(32:32) and (**c**) ACQ(42:24). (**d**–**f**): Lateral profiles obtained from the simulated images at different depths, for the three cases.

If the performance is tested against a simulated phantom containing several reflectors ( Figure 12), small differences between the strategies are highlighted. Although both proposed acquisition strategies increase the lateral resolution respect to the TFM, they also show higher sidelobes. To analyse how the lateral resolution has been increased, we have presented lateral profiles at different depths in Figure 12d–f. If both strategies are compared, the ACQ(32:32) shows lower sidelobes than the ACQ(42:24). To obtain a numerical value for this observation, we have used the mean of the ratio (pixel-by-pixel) between the normalized image obtained by the normalised ACQ image and the normalized FMC image that is considered as the reference:

(8) $$R_{ACQ}=E\left[\frac{im_{ACQ}\left(\vec{x}\right)}{im_{FMC}\left(\vec{x}\right)}\right],$$

where $im_{FMC}\left(\vec{x}\right)$ is the image obtained with the FMC and $im_{ACQ}\left(\vec{x}\right)$ is the image obtained with a specific ACQ. By evaluating this ratio on ACQ(32:32) results in $R_{ACQ}=1.72$, and on ACQ(42:24), $R_{ACQ}=1.97$. These values confirm that ACQ(32:32) has lower secondary lobes than ACQ(42:24).

## 6. Conclusions

In this work, we have shown, based on the concepts of the Radon transform and the equivalent linear array, how to study the coarray spatial redundancy of non-grid 2D sparse arrays like spirals or annular rings. Furthermore, we have developed a process to exploit this spatial redundancy to reduce the electronic resources and the computational cost of the beamforming operations of the synthetic aperture system. It has been shown that, with an adequate selection of signals, the resources of an imaging system can be reduced without significant degradation of the original FMC dynamic range. However, the reduction of redundancy makes the coarray shape smoother, which increases the lateral resolution but also increases the secondary lobes.

This procedure improves the capabilities of the system designer to control the performance of sparse arrays based on spirals and other non-grid patterns. Restrictions can be imposed, such as in the number of parallel channels and the number of emission elements. Other conditions, like eliminating specific elements, can also be applied, which could be the case due to malfunctioning transducers and, consequently, this process can be used to design fault-tolerant solutions. Finally, it can provide a measure of the aperture information quality that could be useful for the design/development of new beamforming methods based on sparse coarray reconstruction. In addition, based on the redundancy generated by the reciprocity, a generic solution with an efficient multiplexer design has also been presented.

The proposed methodology provides a solution to developing systems with low resources which can be easily be embedded in complex or autonomous systems. Considering ACQ(64:16), this solution uses only 16 receiver channels, 64 emitters, and one transmitter. Then, according to the standard electronic integration levels of the market, we can design a simple aperture that integrates the electronic front-end close to the transducer, reducing electrical noise and interconnection and communication problems. This opens up the possibility of developing instrumentation integrated into Internet of Things systems for NDT applications.

## Figures and Tables

**Table 1:** Fermat spiral array: performance for the FMC, RCP and all six strategies considered (Tx:Rx).

ACQ	$M_{o}$	$M_{r}$	$N_{s}$	$N_{c}$	$\Delta \theta_{-6\mathrm{dB}}$	CP (dB)	DR (dB)	$\Delta \theta_{\mathrm{DR}}$
FMC	81%	7.23	4096	64	$2.92^{{}^{\circ}}$	-	−35	$11.5^{{}^{\circ}}$
RCP	81%	3.68	2080	33	$2.92^{{}^{\circ}}$	-	−35	$11.5^{{}^{\circ}}$
(64:64)	81%	2.93	1630	32	$2.6^{{}^{\circ}}$	−20.9	−35.4	$15.1^{{}^{\circ}}$
(64:32)	81%	2.93	1631	32	$2.6^{{}^{\circ}}$	−20.8	−36	$14.0^{{}^{\circ}}$
(64:16)	77%	2.03	1024	16	$2.3^{{}^{\circ}}$	−20.7	−34	$13.4^{{}^{\circ}}$
(64:8)	59%	1.43	512	8	$2.0^{{}^{\circ}}$	−21.7	−28.6	$11.3^{{}^{\circ}}$
(32:64)	77%	2.71	1444	50	$2.6^{{}^{\circ}}$	−21	−33.8	$12.0^{{}^{\circ}}$
(16:64)	65%	2.02	984	60	$2.6^{{}^{\circ}}$	−18	−31.9	$12.8^{{}^{\circ}}$

**Table 2:** Segmented Annular Array. Performance for (64:64), (64:24), (64:16) and (64:8). Simulated and experimental data included for $\Delta \theta_{-6\mathrm{dB}}$, DR (dB) and $\Delta \theta_{DR}$.

ACQ	$M_{o}$	$M_{r}$	$N_{s}$	$N_{c}$	$\Delta \theta_{-6\mathrm{dB}}$		DR (dB)		$\Delta \theta_{\mathrm{DR}}$	
					Sim.	Exp.	Sim.	Exp.	Sim.	Exp.
(64:64)	66%	1.8	1536	29	$2.80^{{}^{\circ}}$	$3.17^{{}^{\circ}}$	−20.86	−18.91	$6.40^{{}^{\circ}}$	$6.96^{{}^{\circ}}$
(64:24)	60%	1.5	1286	24	$2.81^{{}^{\circ}}$	$3.20^{{}^{\circ}}$	−20.51	−18.59	$6.21^{{}^{\circ}}$	$6.38^{{}^{\circ}}$
(64:16)	52%	1.3	1024	16	$2.82^{{}^{\circ}}$	$3.21^{{}^{\circ}}$	−20.79	−17.41	$6.40^{{}^{\circ}}$	$6.70^{{}^{\circ}}$
(64:8)	35%	1.2	512	8	$2.71^{{}^{\circ}}$	$3.10^{{}^{\circ}}$	−20.59	−18.12	$6.73^{{}^{\circ}}$	$10.02^{{}^{\circ}}$

**Table 3:** Spiral array: FMC, RCP, ACQ(42:24) and ACQ(32:32) acquisition strategies results.

ACQ	$M_{o}$	$M_{r}$	$N_{s}$	$N_{c}$	$\Delta \theta_{-6\mathrm{dB}}$	CP (dB)	DR (dB)	$\Delta \theta_{\mathrm{DR}}$
FMC	81%	7.23	4096	64	$2.92^{{}^{\circ}}$	-	−35	$11.5^{{}^{\circ}}$
midrule RCP	81%	3.68	2080	33	$2.92^{{}^{\circ}}$	-	−35	$11.5^{{}^{\circ}}$
(42:24)	75%	2.02	1008	24	$2.34^{{}^{\circ}}$	−21	−33.4	$12.9^{{}^{\circ}}$
(32:32)	74%	2.05	1024	32	$2.42^{{}^{\circ}}$	−20.6	−33.11	$14^{{}^{\circ}}$
